# Healthy or misleading? A study of food outlets categorised as ‘healthy’ on an Australian delivery platform

**DOI:** 10.1017/S1368980026102997

**Published:** 2026-07-06

**Authors:** Rebecca Bennett, Christina Zorbas, Adyya Gupta, Laura Veronica Alston, Sachin Wasnik, Cindy Needham

**Affiliations:** 1 Global Centre for Preventive Health and Nutrition, Institute for Health Transformation, Deakin University, Geelong, Australia; 2 Deakin Rural Health, School of Medicine, Deakin University, Warrnambool, Australia; 3 Research Unit, Colac Area Health, Colac, Australia; 4 Deakin University, Geelong, Australia

**Keywords:** Food environment, Online food delivery, Online food retail, Australia

## Abstract

**Objective:**

Online food delivery (OFD) platforms increasingly shape food environments and dietary choices, yet it remains unclear whether platform-defined ‘healthy’ outlet categories align with evidence-informed assessments. This study assessed the healthiness of food outlets labelled as ‘healthy’ on a leading OFD platform in Victoria, Australia, using food environment classification tools.

**Design:**

Cross-sectional study using web-scraped outlet-level data. Outlets labelled ‘healthy’ were assigned to one of thirty-six predefined outlet types and classified using the DIGIASSESS index, an expert-informed food environment scoring tool. A supplementary menu-based sensitivity analysis was conducted in a stratified random 10 % subsample of outlets.

**Setting:**

A leading OFD platform in Victoria, Australia.

**Participants:**

We identified 12 938 unique food outlets, of which 1408 (9·2 %) were labelled ‘healthy’ by the platform and included in the primary analysis. A stratified random subsample of 166 outlets underwent menu-level review.

**Results:**

‘Healthy’-labelled outlets were most commonly Independent–Takeaway (11·8 %), Independent–Cereal-Based Café Meals (9·7 %) and Service Station Convenience Stores (7·4 %). Using DIGIASSESS, most ‘healthy’-labelled outlets were reclassified as ‘less healthy’ (*n* 1123; 79·7 %) or ‘unhealthy’ (*n* 180; 12·8 %), with 106 (7·5 %) classified as ‘healthy.’ The supplementary menu-based analysis showed similar classifications to DIGIASSESS (> 98 % agreement).

**Conclusions:**

On this leading Australian OFD platform, the ‘healthy’ outlet category did not align with expert-informed assessments of outlet-type healthiness. Such misalignment risks misleading consumers and highlights the need for transparent, standardised criteria governing health-related outlet categories in digital food environments.

Unhealthy diets, including those high in saturated fats, added sugars and Na, are a leading risk factor for non-communicable diseases including CVD, type 2 diabetes, some cancers, as well as development of overweight and obesity^(1)^. Globally, in 2015, overweight and obesity contributed to 4 million deaths and 120 million disability-adjusted life years^(2)^.

When making food purchasing decisions, consumers rely on a range of information sources to determine the nutritional content and suitability of products to meet their requirements and preferences. These can include nutritional information panels, descriptions from packaging or menus and claims made by food manufacturers^(3)^. However, food industry marketing tactics can often misinform consumers^(4,5)^. The accurate presentation of nutritional information can influence consumer perceptions and choices, particularly when health-related claims are used^(6,7)^.

Increasingly, consumers are purchasing food and drinks from online food delivery (OFD) platforms^(8)^. These platforms provide consumers with a convenient way to order meals from fast-food outlets, restaurants and cafes, as well as items from convenience stores and supermarkets^(9)^. An analysis of meals purchased away from home in five countries between 2018 and 2021 found that while the overall number of meals purchased out-of-home remained consistent, the proportion ordered using an OFD platform almost doubled^(10)^. Additionally, analysis of nationally representative survey data found that 34 % of Australians reported using an OFD platform to order meals in the previous 30 d^(11)^. Other recent studies have shown that the majority of foods available on OFD platforms are unhealthy^(12,13)^, and that unhealthy food outlets are more common in areas of socio-economic disadvantage^(14,15)^ and regional areas (population centres outside major metropolitan areas)^(15)^. Despite this, OFD platforms often use an unregulated ‘healthy’ category label for food outlets within their apps. However, there is limited evidence regarding the validity of these classifications and whether they support or hinder informed consumer choice and public health nutrition efforts. This study aimed to examine how a leading OFD platform classifies ‘healthy’ food outlets in Australia compared to classification by expert-informed proxy tools for assessing the healthiness of online food outlets.

## Methods

### Classification of food outlet categories on OFD platforms

Information about how retailers select outlet categories was obtained through direct observation of the platform’s publicly accessible merchant onboarding interface during the study period. During registration, retailers select a business type and one or more platform-defined cuisine/outlet categories (including a ‘Healthy’ category). Food outlets are able to select more than one category. Cuisine categories were captured as unordered fields within the scraped dataset, meaning it was possible to identify whether ‘Healthy’ had been selected, but not whether it was entered as a primary or secondary category. Category ordering is not visible within the consumer-facing platform interface. Accordingly, analyses focused on all outlets surfaced within the platform-defined ‘Healthy’ category, and outlets were included if they had selected ‘Healthy’ as one of their nominated categories.

### Data collection

Food outlet data were collected from the publicly accessible Australian website of a leading OFD platform operating in Victoria, Australia. The platform name is not disclosed in this manuscript; however, at the time of data collection (November 2022–January 2023), the platform accounted for a substantial share of Australian OFD transactions^(16)^. Data were collected using web scraping methods between November 2022 and January 2023, using an automated script written in Python. Collected data included outlet-level attributes such as food outlet category, food outlet name, delivery time and fee and suburb name and postal code. The script ran between 11.00 and 22.00 to account for variation in outlet operating hours. During pilot testing of the web scraping script, data collection errors occurred when outlets were closed, resulting in missing or incomplete information such as delivery times and fees. To minimise this issue and ensure completeness of outlet-level data, the script was run repeatedly during typical operating hours across multiple days.

Data collection did not rely on individual street addresses. Instead, web scraping queries were conducted using all Victorian postal codes, allowing comprehensive capture of outlets available across metropolitan, regional and rural areas of Victoria.

### Data analysis

Food outlets labelled as ‘healthy’ were extracted from the dataset and checked for duplicates. As consistent nutrient-level data were not available on OFD platforms, we used two complementary proxy approaches to assess outlet healthiness: the DIGIASSESS index, based on expert consensus, and a supplementary menu-level analysis adapted from Goffe *et al.* ^(9,17)^. DIGIASSESS applies to a broad range of outlet types available on online delivery platforms, including ready-to-eat food outlets as well as food retailers such as supermarkets and convenience stores. These outlets were included because online delivery platforms increasingly facilitate food purchasing across multiple retail formats, making them relevant components of the digital food environment.

The DIGIASSESS index is an expert-informed digital food environment scoring tool developed to classify the relative healthiness of food outlet types commonly available on OFD platforms. During development of the DIGIASSESS index, Australian dietitians and public health nutrition experts were provided with sample menus representing each of the thirty-six outlet types commonly available on online delivery platforms. Experts independently assessed these menus and assigned a score between –10 (least healthy) and +10 (most healthy) based on the overall nutritional profile and typical menu composition. Scoring considerations could include the relative predominance of discretionary *v*. core food groups, the frequency of fried or energy-dense items, the availability of vegetables and minimally processed options and overall alignment with the Australian Dietary Guidelines^(18)^. The final DIGIASSESS score for each outlet type reflects expert consensus across these assessments. A sample outlet menu provided to participants to assist them to determine their food environment score is available in online supplementary material, Supplemental Table 1.

Importantly, DIGIASSESS assigns scores at the outlet-type level rather than assessing the nutrient content of individual menu items. It therefore provides a proxy measure of food environment healthiness rather than a direct nutritional assessment.

During tool development, the top twenty fast-food and café franchises were identified using the Australian Franchise Registry^(19)^ and categorised using keyword matching. Remaining outlets that were listed in multiple locations by the same name were also searched to confirm whether they were a franchise.

Food outlet types scoring less than –5 were classified as ‘unhealthy’ (e.g. liquor stores, franchise pizza restaurants and convenience stores), those scoring between –5 and +5 were classified as ‘less healthy’ (e.g. independent takeaway shops, franchise burger restaurants and supermarkets) and those scoring +5 or greater were classified as ‘healthy’ (e.g. sushi restaurants and greengrocers), following previous food environment literature^(9,20,21)^. For example, ‘Independent – Takeaway’ referred to independently operated fish and chip shops, pizza shops, charcoal chicken outlets, kebab shops and burger outlets that were not part of a franchise. Several outlet types within the ‘less healthy’ category (e.g. Independent – cereal-based café meals; Independent – mixed vegetable and meat dishes; Franchise – salads) may also include individual menu items of varying nutritional quality; however, classifications reflect dominant menu patterns typically observed on online delivery platforms rather than the healthiest options available within those outlets. DIGIASSESS classifications were based on expert assessment of representative menus and reflected the overall composition of foods typically available within each outlet type, rather than isolated menu items. Accordingly, the presence of some healthier options within an outlet did not necessarily alter its classification if the dominant menu pattern remained characterised by discretionary or energy-dense foods. During expert assessment, distinctions between franchise and independently operated outlet types were retained where menu composition and broader food environment characteristics differed systematically.

In the present study, outlets were classified into these predefined outlet types using automated keyword matching and manual verification. No individual menu items were scored using DIGIASSESS; rather, the tool assigns healthiness scores at the outlet-type level based on prior expert evaluation. Automated classification used validated keyword matching, based on outlet names (e.g. an outlet called ‘Melbourne Sushi’ would be automatically classified as ‘sushi’)^(15)^. Manual classification was conducted by the research team for outlets with names containing keywords from more than one category (e.g. ‘Bakery Café’ containing both keywords for the ‘Bakery’ category and for the ‘Independent – cereal-based café meals’ category). This involved manual review of the outlet’s menu by the research team to assign the category that best reflected its dominant menu offerings.

### Supplementary analysis

A supplementary menu-based sensitivity analysis, adapted from Goffe *et al.* ^(17)^, was conducted between January and May 2026 to examine whether outlet classifications were consistent with observable menu-level features. Although DIGIASSESS has previously been developed and published^(9)^, it operates at the outlet-type level. In the absence of comprehensive nutrient profiling, the sensitivity analysis allowed us to explore potential within-category variation and assess whether menu-level features were broadly consistent with outlet-type classifications.

Given the manual and resource-intensive nature of menu review, and the large size of the overall dataset (1408 outlets labelled as ‘healthy’), this assessment was applied to a proportionate stratified random subsample (10 % within each DIGIASSESS category). Stratification ensured representation across outlet types while maintaining feasibility. Where a selected outlet was no longer operating or its menu was no longer publicly accessible at the time of review, a replacement outlet from the same DIGIASSESS category was randomly selected.

According to Goffe *et al.*, each outlet was assessed using a scoring tool based on five criteria:

1. the presence of healthy menu options (e.g. salads and grilled items),
2. use of healthy cooking methods (e.g. poaching, steaming, grilling and baking),
3. the presence of nutritional labelling (e.g. kilojoule/energy labels),
4. point-of-sale information promoting health (e.g. wording such as ‘healthy’ or ‘fresh’),
5. availability of healthy beverages (e.g. water, sparkling water and no-added-sugar juices).

Healthy cooking methods were only recorded where preparation techniques (e.g. grilled, baked, steamed or poached) were explicitly described within menu text. No assumptions were made when cooking methods were not specified, and menu images were not used to infer preparation techniques.

Each criterion was scored from 0 to 2 points depending on its presence or absence, yielding a maximum score of 10 per outlet. Scores were categorised as follows: 0–2 = ‘unhealthy’, 3–5 = ‘less healthy’ and 6–10 = ‘healthy’. These results were then compared to DIGIASSESS classifications to explore alignment between the two tools. While this analysis provides a more granular, menu-level perspective, it remains a proxy measure given the absence of consistent nutrient-level data on OFD platforms.

Descriptive statistics were used to calculate the overall frequency of food outlets according to the DIGIASSESS healthiness categories (unhealthy, less healthy and healthy) and outlet types.

## Results

The web scrape resulted in the identification of 12 938 unique food outlets, of which 1408 were classified as healthy by the platform (9·18 %). Most outlets within the ‘Healthy’ category (78 %) selected either only the ‘Healthy’ category or ‘Healthy’ plus one additional category. Outlets categorised as ‘healthy’ by the platform were most commonly Independent–Takeaway (*n* 166, 11·8 %), Independent–Cereal-Based Café Meals (*n* 136, 9·7 %) and Service Station Convenience Stores (*n* 104, 7·4 %). These outlets were subsequently reclassified using DIGIASSESS, with the majority classified as ‘less healthy’ (79·7 %) or ‘unhealthy’ (12·8 %).

Results from the menu-based analysis (*n* 166) showed high concordance (> 98 %), suggesting that both proxy approaches to nutrition assessment captured similar classification patterns across outlet types. Outlet types with discrepant classifications were limited to greengrocers (0·43 % of the total sample) and butchers (0·28 %). Detailed results of the menu-based assessment are presented in online supplementary material, Supplemental Table 2, and the comparison between DIGIASSESS and the menu-based classifications is shown in online supplementary material, Supplemental Table 3.

## Discussion

This study provides one of the first empirical examinations of how OFD platforms categorise and promote outlets labelled as ‘healthy’. Using two complementary proxy approaches, we identified systematic misclassification within a leading Australian OFD platform’s ‘healthy’ category.

Our analysis found that, when reclassified using DIGIASSESS, most outlets within the ‘healthy’ category were ‘less healthy’ (79·70 %, *n* 1123), and the most common outlet type was ‘Independent – takeaway’ (*n* 166, 11·78 %) (Table 1). This suggests that outlets explicitly labelled as ‘healthy’ by the platform may nonetheless predominantly offer discretionary or energy-dense menu items. This contribution extends existing literature, which has largely focused on the overall healthiness of foods available or promoted on OFD platforms, by demonstrating misclassification at the outlet level itself.

**Table 1.** Classification of outlets from the ‘Healthy’ category from a leading online food delivery platform in Victoria, Australia, using the DIGIASSESS index adapted from Bennett *et al.* ^(9)^ Table 1 long description.

Food outlet type	*n*, %	Healthiness classification (DIGIASSESS index)
Independent – takeaway	166, 11·79	Less healthy
Independent – cereal-based ‘café’ meals	136, 9·66	Less healthy
Service station convenience stores	104, 7·39	Unhealthy
Independent – noodles with deep-fried items	92, 6·53	Less healthy
Independent – noodles without deep-fried items	84, 5·97	Less healthy
Independent – drinks	81, 5·75	Less healthy
Independent – salads	72, 5·11	Healthy
Franchise – sandwich	54, 3·84	Less healthy
Minor supermarket	51, 3·62	Less healthy
Independent – meat mixed meals with fried potatoes	50, 3·55	Less healthy
Franchise – burgers	47, 3·34	Less healthy
Independent – cereal-based ‘restaurant’ meals	44, 3·13	Less healthy
Franchise – taco	42, 2·98	Less healthy
Independent – desserts	35, 2·48	Unhealthy
Franchise – bakery	34, 2·41	Less healthy
Franchise – salads	33, 2·34	Less healthy
Franchise – chicken	29, 2·06	Less healthy
Convenience stores	27, 1·92	Unhealthy
Franchise – drinks	26, 1·85	Less healthy
Independent – sandwich	25, 1·78	Less healthy
Sushi	24, 1·70	Healthy
Major supermarket	23, 1·63	Less healthy
Bakery	21, 1·49	Less healthy
Independent – curry	21, 1·49	Less healthy
Franchise – noodles	19, 1·35	Less healthy
Independent – mixed vegetable and meat dishes	18, 1·28	Less healthy
Franchise – ready-to-eat microwave meals	15, 1·07	Less healthy
Franchise – desserts	14, 0·99	Unhealthy
Pub	9, 0·64	Less healthy
Greengrocer	6, 0·43	Healthy
Butcher/seafood	4, 0·28	Healthy
Franchise – coffee café	2, 0·14	Less healthy

To provide additional contextual insight, we conducted a supplementary menu-based sensitivity analysis adapted from Goffe *et al.* ^(17)^. While high concordance (> 98 %) was observed between the two approaches, this should not be interpreted as formal validation of DIGIASSESS. Rather, it indicates that both proxy approaches identified similar patterns of outlet-type classification within the constraints of currently available data. The menu-based analysis offered insight into observable menu features within outlet types, whereas DIGIASSESS provides a scalable tool for large digital food environment assessments. These approaches therefore serve complementary rather than confirmatory roles. Together, these findings reinforce existing evidence that OFD platforms predominantly feature outlet types associated with less healthy food environments^(12,13)^. They also highlight a structural limitation in digital food environment research: the absence of consistent, accessible nutritional information at scale. In this context, proxy approaches remain necessary, while underscoring the need for more transparent and standardised systems to evaluate menu healthiness across OFD platforms.

A central implication of these findings relates to regulatory oversight of outlet categorisation on OFD platforms. Currently, retailers can self-select into platform-defined categories such as ‘healthy’ without transparent eligibility criteria or independent verification. This creates the potential for inconsistent application of health-related labels and consumer misunderstanding. Regulatory approaches could require platforms to define and publicly disclose eligibility criteria for health-related categories, implement verification processes and adhere to standardised benchmarks for inclusion. Where health-related categories function as consumer guidance tools, they should be subject to comparable standards of transparency and accountability that align with other forms of health-related marketing claims. Existing nutrition policies, such as mandatory kilojoule labelling, demonstrate that regulation can extend to online food retail settings; however, documented compliance gaps suggest that monitoring and enforcement mechanisms remain insufficient^(22)^. Strengthening oversight of both menu labelling compliance and platform-defined health categories would help reduce misleading cues in digital food environments. A ‘health halo’ effect can occur when foods are marketed as healthy through their description or presentation, leading consumers to underestimate energy content and overconsume^(23)^. By promoting food outlets as healthy, OFD platforms may be creating health halos and misleading consumers into believing they are making healthier choices and hindering their ability to make informed food choices.

To our knowledge, there is no widely adopted, scalable tool capable of evaluating the diverse and dynamic food offerings across OFD platforms. Future research could seek to combine menu-level nutritional profiling tools with platform-level monitoring approaches to support both outlet-level reformulation and broader platform accountability. Monitoring compliance with such standards should ideally fall under the purview of public health authorities, particularly if regulations evolve to better address digital food environments. Artificial intelligence systems developed by researchers have the potential to assist with menu assessments at scale to monitor platform and ensure delivery platforms comply. However, there remains a place for methods that are able to be used to evaluate food environments through human classification alone, in a feasible way.

This study has several limitations. First, due to the large scale of digital food environments, DIGIASSESS classifies outlet healthiness at the outlet-type level based on expert consensus regarding typical menu composition, rather than assessing the nutrient content of individual menu items. As such, it does not capture within-outlet variability, reformulation efforts or the presence of healthier options within outlets classified as ‘less healthy’. Individual menu items within these outlets may align with dietary guidelines, even if the dominant menu pattern does not. Accordingly, classification of outlet types should not be interpreted as equivalent to comprehensive nutritional assessment of all individual menu items offered within those outlets. Second, although expert scoring during DIGIASSESS development was informed by representative sample menus, the index remains a proxy measure and cannot substitute for direct nutrient profiling. More detailed menu-level assessment was constrained by the absence of consistent, accessible nutritional information on OFD platforms, limiting the feasibility of conducting objective item-level nutrient assessment at scale. Additionally, while the primary web scrape was conducted between November 2022 and January 2023, the supplementary menu-level sensitivity analysis was conducted between January and May 2026. As menu offerings may have changed over time, the supplementary analysis should be interpreted as a contextual assessment of menu patterns rather than a direct contemporaneous validation of outlet classifications. Finally, expanding menu-level assessment to the full sample would have required extensive manual coding of large and dynamic menus across 1408 ‘healthy’-labelled outlets and was not feasible within the scope of this study. While recent studies^(24,25)^ have employed artificial intelligence-assisted approaches to evaluate menu healthiness at scale, these methods require additional data access and computational resources that were not available for this study. Future research should continue to explore methods that integrate scalable outlet-level classification with more detailed menu-level nutritional assessment.

### Conclusion

Most food outlets labelled as ‘healthy’ on a leading OFD platform in Victoria were classified as less healthy or unhealthy when assessed using an expert-informed food environment index. This misalignment suggests that platform-defined health categories may function as misleading signals rather than reliable consumer guidance. In the absence of consistent, accessible nutrient-level information, digital food environment research must rely on proxy tools; however, responsibility for accurate health-related categorisation should not rest solely with researchers. Clear eligibility criteria, transparent classification processes and strengthened oversight of health-related labels on OFD platforms are needed to improve accountability in digital food environments.

## Supporting information

10.1017/S1368980026102997.sm001 Bennett et al. supplementary material Bennett et al. supplementary material

## Data Availability

The datasets analysed during the current study are available from the corresponding author on reasonable request.

## Supplementary material

For supplementary material accompanying this paper visit https://doi.org/10.1017/S1368980026102997

## Table 1. Long description

The table categorizes various food outlets based on their healthiness classification using the DIGIASSESS index. It has 3 columns and 28 rows. The columns are labeled ‘Food outlet type’, ‘n, %’, and ‘Healthiness classification (DIGIASSESS index)’. The table lists different types of food outlets along with their respective counts, percentages, and healthiness classifications. Notable entries include ‘Independent takeaway’ with 166 outlets (11.79 percent) classified as less healthy, ‘Service station convenience stores’ with 104 outlets (7.39 percent) classified as unhealthy, and ‘Independent salads’ with 72 outlets (5.11 percent) classified as healthy. The table also includes various franchise and independent outlets with their respective healthiness classifications.

Navigate back to Table 1..
