# Supplementary material for: Healthy or misleading? A study of food outlets categorised as ‘healthy’ on an Australian delivery platform
Source: Public Health Nutr. 2026 Jul 6;29(1):e150. doi: 10.1017/S1368980026102997 (PMC13434493; doi:10.1017/S1368980026102997)
Supplement: Supplementary file 1 [file S1368980026102997sup001.docx]

Supplementary Table 1: An example of a food outlet menu provided to expert participants when determining their food environment score for DIGIASSESS

| Independent - takeaway | Cereal-based mixed meal | Pizzas, burgers, wraps, kebabs/gyros, pita bread, fried dim sims, fried onion rings, pasta dishes |
| --- | --- | --- |
|  | Vegetable-based mixed meal | Salads (coleslaw, garden salad, etc), scalloped potatoes (potatoes, cream and cheese), |
|  | Meat or alternative based meal | Charcoal chicken, grilled fish, grilled meats, battered fish, chicken schnitzels, |
|  | Fried potato | Potato fries, potato cakes, sweet potato chips |
|  | Baked good/Desserts | Baklava, pudding, ice creams |
|  | Sugar Sweetened Beverages | Soft drinks, sports drinks, fruit juice, iced teas |
|  | Non-sugar Sweetened Beverages | Diet soft drinks |

Supplementary Table 2: Nutrition assessment of menus from different outlets using criteria adapted from Goffe et al (1)

| Category | Outlet | Healthy options available^1^ | Uses healthy cooking methods^2^ | Provides nutritional information for menu items^3^ | Point-of-sale information related to health^4^ | Healthy beverages available^5^ | Total score^6^ |
| --- | --- | --- | --- | --- | --- | --- | --- |
| Independent – takeaway | 1 | 2 | 1 | 0 | 0 | 1 | 4 |
|  | 2 | 1 | 0 | 0 | 0 | 1 | 2 |
|  | 3 | 2 | 1 | 0 | 0 | 1 | 4 |
|  | 4 | 1 | 0 | 0 | 0 | 1 | 2 |
|  | 5 | 0 | 0 | 1 | 0 | 1 | 2 |
|  | 6 | 1 | 1 | 0 | 0 | 1 | 3 |
|  | 7 | 0 | 0 | 0 | 0 | 0 | 0 |
|  | 8 | 2 | 1 | 0 | 0 | 1 | 4 |
|  | 9 | 0 | 0 | 0 | 0 | 0 | 0 |
|  | 10 | 1 | 0 | 0 | 0 | 1 | 2 |
|  | 11 | 2 | 0 | 0 | 0 | 2 | 4 |
|  | 12 | 2 | 0 | 0 | 0 | 2 | 4 |
|  | 13 | 2 | 1 | 0 | 0 | 2 | 5 |
|  | 14 | 0 | 0 | 0 | 0 | 0 | 0 |
|  | 15 | 2 | 1 | 0 | 0 | 0 | 3 |
|  | 16 | 2 | 1 | 0 | 0 | 1 | 4 |
| Independent - cereal based “café” meals | 1 | 2 | 1 | 0 | 0 | 1 | 4 |
|  | 2 | 1 | 1 | 0 | 0 | 2 | 4 |
|  | 3 | 1 | 1 | 0 | 0 | 1 | 3 |
|  | 4 | 1 | 1 | 0 | 0 | 1 | 3 |
|  | 5 | 2 | 1 | 0 | 0 | 1 | 4 |
|  | 6 | 2 | 1 | 0 | 0 | 1 | 4 |
|  | 7 | 2 | 2 | 0 | 0 | 2 | 6 |
|  | 8 | 2 | 1 | 0 | 0 | 2 | 5 |
|  | 9 | 2 | 1 | 0 | 1 | 0 | 4 |
|  | 10 | 2 | 1 | 0 | 0 | 1 | 4 |
|  | 11 | 2 | 0 | 0 | 1 | 0 | 3 |
|  | 12 | 1 | 1 | 0 | 0 | 0 | 2 |
|  | 13 | 1 | 1 | 0 | 1 | 2 | 5 |
|  | 14 | 2 | 1 | 0 | 0 | 0 | 3 |
| Service station convenience stores | 1 | 1 | 0 | 0 | 0 | 1 | 2 |
|  | 2 | 1 | 0 | 0 | 0 | 2 | 3 |
|  | 3 | 1 | 0 | 0 | 0 | 1 | 2 |
|  | 4 | 1 | 0 | 0 | 0 | 1 | 2 |
|  | 5 | 1 | 0 | 0 | 0 | 2 | 3 |
|  | 6 | 0 | 0 | 0 | 0 | 2 | 2 |
|  | 7 | 1 | 0 | 0 | 0 | 2 | 3 |
|  | 8 | 1 | 0 | 0 | 0 | 2 | 3 |
|  | 9 | 1 | 0 | 0 | 0 | 2 | 3 |
|  | 10 | 1 | 0 | 0 | 0 | 1 | 2 |
|  | 11 | 1 | 0 | 0 | 0 | 1 | 2 |
| Independent - noodles with deep fried items | 1 | 2 | 1 | 0 | 0 | 0 | 3 |
|  | 2 | 2 | 1 | 0 | 0 | 0 | 3 |
|  | 3 | 2 | 1 | 0 | 0 | 1 | 4 |
|  | 4 | 2 | 1 | 0 | 0 | 0 | 3 |
|  | 5 | 1 | 0 | 0 | 0 | 2 | 3 |
|  | 6 | 1 | 1 | 0 | 0 | 1 | 3 |
|  | 7 | 2 | 1 | 0 | 0 | 1 | 4 |
|  | 8 | 2 | 1 | 0 | 0 | 1 | 4 |
|  | 9 | 2 | 2 | 0 | 0 | 2 | 6 |
|  | 10 | 2 | 1 | 0 | 0 | 0 | 3 |
| Independent - noodles without deep fried items | 1 | 2 | 2 | 0 | 0 | 1 | 5 |
|  | 2 | 2 | 1 | 0 | 0 | 2 | 5 |
|  | 3 | 2 | 2 | 0 | 1 | 0 | 5 |
|  | 4 | 2 | 1 | 0 | 0 | 2 | 5 |
|  | 5 | 2 | 2 | 0 | 0 | 1 | 5 |
|  | 6 | 2 | 2 | 0 | 1 | 2 | 7 |
|  | 7 | 2 | 1 | 0 | 0 | 0 | 3 |
|  | 8 | 2 | 0 | 0 | 0 | 2 | 4 |
|  | 9 | 2 | 2 | 0 | 0 | 2 | 6 |
| Independent – drinks | 1 | 2 | 1 | 0 | 1 | 1 | 5 |
|  | 2 | 1 | 0 | 0 | 0 | 1 | 2 |
|  | 3 | 1 | 0 | 0 | 1 | 1 | 3 |
|  | 4 | 1 | 0 | 0 | 1 | 1 | 3 |
|  | 5 | 0 | 0 | 0 | 1 | 0 | 1 |
|  | 6 | 2 | 0 | 0 | 0 | 1 | 3 |
|  | 7 | 2 | 0 | 0 | 2 | 0 | 4 |
|  | 8 | 2 | 0 | 0 | 0 | 0 | 2 |
|  | 9 | 2 | 0 | 0 | 0 | 0 | 2 |
| Independent – salads | 1 | 2 | 2 | 0 | 1 | 2 | 7 |
|  | 2 | 2 | 1 | 0 | 1 | 2 | 6 |
|  | 3 | 1 | 1 | 0 | 1 | 2 | 5 |
|  | 4 | 2 | 2 | 0 | 2 | 0 | 6 |
|  | 5 | 2 | 1 | 0 | 2 | 0 | 5 |
|  | 6 | 1 | 1 | 0 | 1 | 2 | 5 |
|  | 7 | 2 | 2 | 0 | 2 | 2 | 8 |
|  | 8 | 2 | 2 | 0 | 0 | 0 | 4 |
| Franchise – sandwich | 1 | 2 | 1 | 0 | 0 | 2 | 5 |
|  | 2 | 2 | 1 | 0 | 0 | 2 | 5 |
|  | 3 | 2 | 1 | 0 | 0 | 2 | 5 |
|  | 4 | 1 | 0 | 0 | 0 | 2 | 3 |
|  | 5 | 2 | 1 | 0 | 0 | 2 | 5 |
|  | 6 | 2 | 1 | 0 | 0 | 2 | 5 |
| Minor supermarket | 1 | 2 | 0 | 0 | 0 | 2 | 4 |
|  | 2 | 2 | 0 | 0 | 0 | 2 | 4 |
|  | 3 | 2 | 0 | 0 | 0 | 2 | 4 |
|  | 4 | 2 | 0 | 0 | 0 | 2 | 4 |
|  | 5 | 2 | 0 | 0 | 0 | 2 | 4 |
|  | 6 | 2 | 0 | 0 | 0 | 2 | 4 |
| Independent - meat mixed meals with fried potatoes | 1 | 1 | 1 | 0 | 0 | 2 | 4 |
|  | 2 | 2 | 1 | 0 | 0 | 1 | 4 |
|  | 3 | 2 | 1 | 0 | 0 | 1 | 4 |
|  | 4 | 2 | 2 | 0 | 0 | 2 | 6 |
|  | 5 | 2 | 1 | 0 | 0 | 2 | 5 |
|  | 6 | 2 | 0 | 0 | 0 | 2 | 4 |
| Franchise – burgers | 1 | 1 | 1 | 2 | 0 | 1 | 5 |
|  | 2 | 0 | 1 | 2 | 0 | 1 | 4 |
|  | 3 | 0 | 1 | 2 | 0 | 1 | 4 |
|  | 4 | 0 | 2 | 2 | 0 | 1 | 5 |
|  | 5 | 0 | 2 | 2 | 0 | 1 | 5 |
| Independent - cereal based “restaurant” meals | 1 | 2 | 1 | 0 | 0 | 1 | 4 |
|  | 2 | 2 | 1 | 0 | 0 | 1 | 4 |
|  | 3 | 2 | 2 | 0 | 0 | 1 | 5 |
|  | 4 | 2 | 2 | 0 | 0 | 2 | 6 |
|  | 5 | 2 | 1 | 0 | 0 | 2 | 5 |
| Franchise – taco | 1 | 1 | 1 | 0 | 1 | 2 | 5 |
|  | 2 | 1 | 1 | 0 | 0 | 2 | 4 |
|  | 3 | 2 | 1 | 2 | 0 | 0 | 5 |
|  | 4 | 1 | 1 | 0 | 0 | 2 | 4 |
|  | 5 | 1 | 0 | 0 | 0 | 2 | 3 |
| Independent – desserts | 1 | 0 | 0 | 0 | 0 | 1 | 1 |
|  | 2 | 0 | 0 | 0 | 0 | 0 | 0 |
|  | 3 | 0 | 0 | 0 | 0 | 0 | 0 |
|  | 4 | 0 | 0 | 0 | 0 | 0 | 0 |
| Franchise – bakery | 1 | 1 | 0 | 0 | 0 | 1 | 2 |
|  | 2 | 2 | 0 | 0 | 0 | 1 | 3 |
|  | 3 | 1 | 0 | 0 | 1 | 2 | 4 |
|  | 4 | 0 | 0 | 0 | 0 | 2 | 2 |
| Franchise – salads | 1 | 2 | 1 | 0 | 0 | 0 | 3 |
|  | 2 | 2 | 1 | 0 | 0 | 2 | 5 |
|  | 3 | 2 | 2 | 2 | 0 | 2 | 8 |
|  | 4 | 2 | 0 | 0 | 0 | 2 | 4 |
| Franchise – chicken | 1 | 0 | 0 | 2 | 0 | 2 | 4 |
|  | 2 | 1 | 1 | 1 | 0 | 2 | 5 |
|  | 3 | 2 | 0 | 1 | 1 | 2 | 6 |
| Convenience stores | 1 | 1 | 0 | 0 | 0 | 2 | 3 |
|  | 2 | 0 | 0 | 0 | 0 | 1 | 1 |
|  | 3 | 0 | 0 | 0 | 0 | 1 | 2 |
| Franchise – drinks | 1 | 1 | 0 | 2 | 1 | 1 | 5 |
|  | 2 | 1 | 0 | 0 | 1 | 0 | 2 |
|  | 3 | 1 | 0 | 2 | 1 | 1 | 5 |
| Independent – sandwich | 1 | 2 | 1 | 0 | 1 | 1 | 5 |
|  | 2 | 2 | 2 | 0 | 0 | 2 | 6 |
|  | 3 | 2 | 0 | 0 | 0 | 1 | 3 |
| Sushi | 1 | 2 | 1 | 2 | 0 | 2 | 7 |
|  | 2 | 2 | 1 | 2 | 0 | 2 | 7 |
|  | 3 | 2 | 2 | 0 | 0 | 2 | 6 |
| Major supermarket | 1 | 2 | 0 | 0 | 1 | 2 | 5 |
|  | 2 | 2 | 0 | 0 | 1 | 2 | 5 |
|  | 3 | 2 | 0 | 0 | 1 | 2 | 5 |
| Bakery | 1 | 2 | 0 | 0 | 0 | 1 | 3 |
|  | 2 | 2 | 1 | 0 | 0 | 2 | 5 |
|  | 3 | 0 | 0 | 0 | 0 | 2 | 2 |
| Independent – curry | 1 | 2 | 1 | 0 | 0 | 0 | 3 |
|  | 2 | 1 | 1 | 0 | 0 | 1 | 3 |
|  | 3 | 1 | 0 | 0 | 1 | 0 | 2 |
| Franchise – noodles | 1 | 1 | 1 | 2 | 0 | 1 | 5 |
|  | 2 | 0 | 0 | 2 | 0 | 2 | 4 |
| Independent - mixed vegetable and meat dishes | 1 | 2 | 2 | 0 | 0 | 0 | 4 |
|  | 2 | 2 | 2 | 0 | 0 | 1 | 5 |
| Franchise - ready to eat microwave meals | 1 | 2 | 1 | 0 | 0 | 0 | 3 |
|  | 2 | 2 | 1 | 0 | 1 | 0 | 4 |
| Franchise – desserts | 1 | 0 | 0 | 2 | 0 | 0 | 2 |
|  | 2 | 0 | 0 | 2 | 0 | 2 |  |
| Pub | 1 | 2 | 1 | 0 | 0 | 2 | 5 |
|  | 2 | 1 | 1 | 0 | 1 | 2 | 5 |
| Greengrocer | 1 | 2 | 0 | 0 | 2 | 1 | 5 |
|  | 2 | 2 | 0 | 0 | 2 | 1 | 5 |
| Butcher / seafood | 1 | 2 | 0 | 0 | 2 | 0 | 4 |
|  | 2 | 2 | 0 | 0 | 2 | 0 | 4 |
| Franchise - coffee café | 1 | 1 | 0 | 2 | 0 | 2 | 5 |
|  | 2 | 2 | 0 | 0 | 1 | 2 | 5 |

###### 1: Healthy options available, e.g. salads, grilled options. 0=no options, 1=one option, 2=two or more options

###### 2: Uses healthy cooking methods, e.g. baked, steamed, poached. 0=no healthy methods, 1=one healthy method, 2=two or more healthy methods

###### 3: Provides energy information for menu items. 0=no nutritional information, 1=nutritional information provided for one menu category, 2=nutritional information provided for two or more menu categories

###### 4: Point-of-sale information related to health, e.g. health focused tags, or wording such as "healthy" or "fresh". 0=no information, 1=information for one menu category, 2=information for two or more menu categories

###### 5: Healthy beverages available, e.g. water, sparkling water, no added sugar juice, etc. 0=no healthy options, 1=one healthy option, 2=two or more healthy options

###### 6: Scores 0-2 = unhealthy, 3-5 = less healthy, 6-8= healthy

Supplementary Table 3: Comparison between assessment of healthiness of different types of food outlets using the DIGIASSESS Index and methods adapted from Goffe et al.

| Outlet type | Proportion of healthy category (%) | 10% random sample (n) | Nutritional assessment (mean score) | Category | Category (DIGIASSESS) | Agreement |
| --- | --- | --- | --- | --- | --- | --- |
| Independent – takeaway | 11.79 | 16 | 3 | Less healthy | Less healthy | Yes |
| Independent - cereal based “café” meals | 9.66 | 14 | 4 | Less healthy | Less healthy | Yes |
| Service station convenience stores | 7.39 | 11 | 2 | Unhealthy | Unhealthy | Yes |
| Independent - noodles with deep fried items | 6.53 | 10 | 4 | Less healthy | Less healthy | Yes |
| Independent - noodles without deep fried items | 5.97 | 9 | 5 | Less healthy | Less healthy | Yes |
| Independent – drinks | 5.75 | 9 | 3 | Less healthy | Less healthy | Yes |
| Independent – salads | 5.11 | 8 | 6 | Healthy | Healthy | Yes |
| Franchise – sandwich | 3.84 | 6 | 5 | Less healthy | Less healthy | Yes |
| Minor supermarket | 3.62 | 6 | 4 | Less healthy | Less healthy | Yes |
| Independent - meat mixed meals with fried potatoes | 3.55 | 6 | 5 | Less healthy | Less healthy | Yes |
| Franchise – burgers | 3.34 | 5 | 5 | Less healthy | Less healthy | Yes |
| Independent - cereal based “restaurant” meals | 3.13 | 5 | 5 | Less healthy | Less healthy | Yes |
| Franchise – taco | 2.98 | 5 | 4 | Less healthy | Less healthy | Yes |
| Independent – desserts | 2.48 | 4 | 0 | Unhealthy | Unhealthy | Yes |
| Franchise – bakery | 2.41 | 4 | 3 | Less healthy | Less healthy | Yes |
| Franchise – salads | 2.34 | 4 | 5 | Less healthy | Less healthy | Yes |
| Franchise – chicken | 2.06 | 3 | 5 | Less healthy | Less healthy | Yes |
| Convenience stores | 1.92 | 3 | 2 | Unhealthy | Unhealthy | Yes |
| Franchise – drinks | 1.85 | 3 | 4 | Less healthy | Less healthy | Yes |
| Independent – sandwich | 1.78 | 3 | 5 | Less healthy | Less healthy | Yes |
| Sushi | 1.70 | 3 | 7 | Healthy | Healthy | Yes |
| Major supermarket | 1.63 | 3 | 5 | Less healthy | Less healthy | Yes |
| Bakery | 1.49 | 3 | 3 | Less healthy | Less healthy | Yes |
| Independent – curry | 1.49 | 3 | 3 | Less healthy | Less healthy | Yes |
| Franchise – noodles | 1.35 | 2 | 5 | Less healthy | Less healthy | Yes |
| Independent - mixed vegetable and meat dishes | 1.28 | 2 | 5 | Less healthy | Less healthy | Yes |
| Franchise - ready to eat microwave meals | 1.07 | 2 | 4 | Less healthy | Less healthy | Yes |
| Franchise – desserts | 0.99 | 2 | 3 | Unhealthy | Unhealthy | Yes |
| Pub | 0.64 | 2 | 5 | Less healthy | Less healthy | Yes |
| Greengrocer | 0.43 | 2 | 5 | Less healthy | Healthy | **No** |
| Butcher / seafood | 0.28 | 2 | 4 | Less healthy | Healthy | **No** |
| Franchise - coffee café | 0.14 | 2 | 5 | Less healthy | Less healthy | Yes |

1.	Goffe L, Uwamahoro NS, Dixon CJ, Blain AP, Danielsen J, Kirk D, et al. Supporting a Healthier Takeaway Meal Choice: Creating a Universal Health Rating for Online Takeaway Fast-Food Outlets. Int J Environ Res Public Health. 2020;17(24):9260.
